# Urban lymphatic filariasis in the metropolis of Dar es Salaam, Tanzania

**DOI:** 10.1186/1756-3305-6-286

**Published:** 2013-09-30

**Authors:** Mbutolwe E Mwakitalu, Mwelecele N Malecela, Erling M Pedersen, Franklin W Mosha, Paul E Simonsen

**Affiliations:** 1 National Institute for Medical Research, P.O. Box 9653, Dar es Salaam, Tanzania; 2 Department of Veterinary Disease Biology, Faculty of Health and Medical Sciences, University of Copenhagen, Thorvaldsensvej 57, 1871 Frederiksberg C, Denmark; 3 KCM-College, Tumaini University, P.O. Box 2240, Moshi, Tanzania

**Keywords:** Lymphatic filariasis, Urban, Circulating filarial antigens, Bm14 antibodies, Microfilariae, Clinical manifestations, Vectors, Dar es Salaam, Tanzania

## Abstract

**Background:**

The last decades have seen a considerable increase in urbanization in Sub-Saharan Africa, and it is estimated that over 50% of the population will live in urban areas by 2040. Rapid growth of cities combined with limited economic resources often result in informal settlements and slums with favorable conditions for proliferation of vectors of lymphatic filariasis (LF). In Dar es Salaam, which has grown more than 30 times in population during the past 55 years (4.4 million inhabitants in 2012), previous surveys have indicated high prevalences of LF. This study investigated epidemiological aspects of LF in Dar es Salaam, as a background for planning and implementation of control.

**Methods:**

Six sites with varying distance from the city center (3–30 km) and covering different population densities, socioeconomic characteristics, and water, sewerage and sanitary facilities were selected for the study. Pupils from one public primary school at each site were screened for circulating filarial antigen (CFA; marker of adult worm infection) and antibodies to Bm14 (marker of exposure to transmission). Community members were examined for CFA, microfilariae and chronic manifestations. Structured questionnaires were administered to pupils and heads of community households, and vector surveys were carried out in selected households.

**Results:**

The study indicated that a tremendous decrease in the burden of LF infection had occurred, despite haphazard urbanisation. Contributing factors may be urban malaria control targeting *Anopheles* vectors, short survival time of the numerous *Culex quinquefasciatus* vectors in the urban environment, widespread use of bed nets and other mosquito proofing measures, and mass drug administration (MDA) in 2006 and 2007. Although the level of ongoing transmission was low, the burden of chronic LF disease was still high.

**Conclusions:**

The development has so far been promising, but continued efforts are necessary to ensure elimination of LF as a public health problem. These will include improving the awareness of people about the role of mosquitoes in transmission of LF, more thorough implementation of environmental sanitation to reduce *Cx. quinquefasciatus* breeding, continued MDA to high-risk areas, and set-up of programmes for management of chronic LF disease.

## Background

The world has witnessed a tremendous increase in urbanization in recent decades. This is also the case for Sub-Saharan Africa, where the urban population has grown from 11.2% in 1950 to 36.3% in 2010 and is estimated to reach more than 50% by the year 2040 [1]. The growth is partly due to migration from rural to urban areas and partly to natural increase in the already existing urban population. Many of the rapid growing cities in developing countries are characterized by insufficient basic infrastructure, and the majority of rural migrants end up in informal and unplanned settlements where basic facilities are poor or absent. In 2010, 62% of the urban population in Sub-Saharan Africa was estimated to live in slums, which are urban areas where households lack access to safe water, adequate sanitation, sufficient living space, durable housing and security of tenure [2]. Such conditions provide favorable habitats for breeding of disease vectors and for transmission of many of the so-called neglected tropical diseases, including lymphatic filariasis (LF), schistosomiasis and soil transmitted helminthiases, which can have severe negative consequences for human health [3-6].

Tanzania has also faced rapid urbanization, with much of it happening in Dar es Salaam, the commercial capital. The population of Dar es Salaam has increased more than 30 times during the past 55 years, from 129,000 in 1957 to 4.4 million in 2012 [7]. Dar es Salaam is currently ranked as the 3^rd^ fastest growing city in Africa and the 9^th^ worldwide [8]. More than 65% of the residents in Dar es Salaam live in un-planned and un-serviced settlements, with poor drinking water and sanitation, poor housing, overcrowding and lack of organized solid waste collection [9-11]. Parts of Dar es Salaam experience flooding during the rains, and it is common to observe clogged open drains, ditches with stagnant household and rain water, and poorly serviced cesspits, septic-tanks and latrines, which support prolific breeding of *Culex quinquefasciatus* mosquitoes [10].

Human LF is a disabling and disfiguring disease, which in Sub-Saharan Africa results from infection with the mosquito-borne filarial nematode *Wuchereria bancrofti*[12]. The vectors responsible for transmission of LF in this region are mainly *Anopheles gambiae* and *An. funestus*. However, the relative role of *Cx. quinquefasciatus* has become increasingly important as a vector in coastal East Africa, particularly in urban and semi-urban environments [13-15]. It is estimated that Sub-Saharan Africa has about 50 million cases of LF, being about one third of the global burden [16], and Tanzania is ranked the 3^rd^ country in Africa in terms of people at risk (34 million) and people infected (6 million) [17]. Most studies on LF have focused on rural areas, where the burden of infection and disease is highest. This is also the case for Tanzania, where numerous studies have documented high levels of LF endemicity in the rural coastal zone (e.g. [13,14,18-20]).

Urban LF has been listed as one of the key challenges in the ongoing global efforts to eliminate LF as a public health problem [21]. Human behavior and culture often differ markedly between rural and urban communities, as do environmental factors that support disease transmission, and simply applying rural control strategies to complex urban settings are not likely to be successful. Instead, strategies are necessary that take the specific behavioral and epidemiological conditions in affected urban environments into consideration. The epidemiology of urban LF has mainly been investigated in large cities in Asia and Brazil [22]. Little is known about urban LF in Sub-Saharan Africa, but two small studies suggested a potential for urban transmission of LF in West Africa [23,24]. In Dar es Salaam, located on the East African coast, past surveys and spot checks have documented a high prevalence of LF [25-28], and cases of microfilaraemia and clinical manifestations have frequently been noted in clinics and hospitals, but no detailed epidemiological surveys have been carried out. The present study investigated LF infection, disease and transmission in the metropolis of Dar es Salaam, as a background for planning and implementation of control.

## Methods

### Study sites and study populations

**Figure 1:**
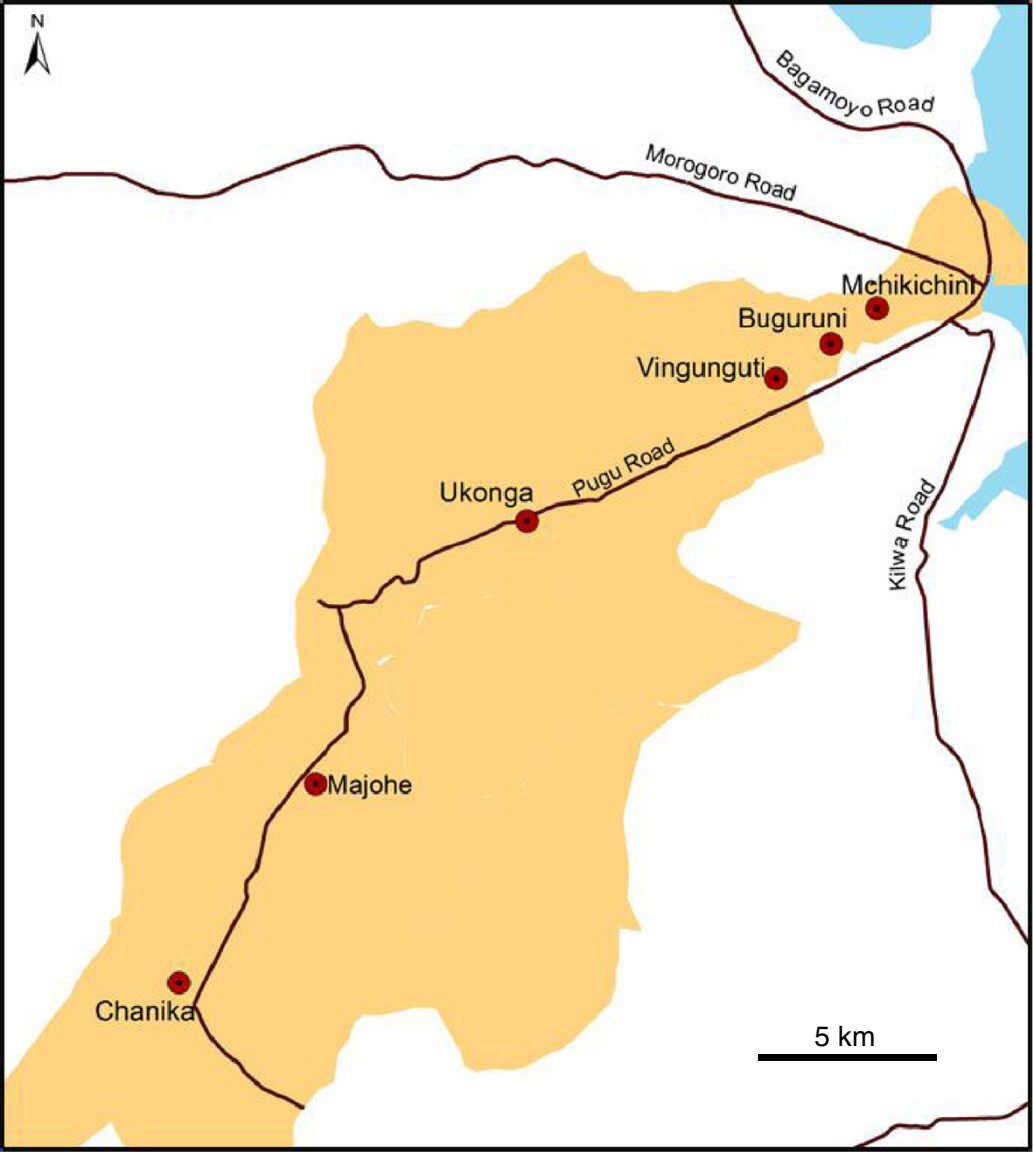
**Map of Dar es Salaam, showing the location of the study schools in the six wards of Ilala District.** Yellow area = Ilala District, Red circles = schools.

Dar es Salaam, located in the eastern part of Tanzania along the Indian Ocean coast, is the largest city and the major commercial center in Tanzania. It has an official population of about 4.4 million (2012 census) and is divided into three districts namely Kinondoni, Ilala and Temeke. The present study was carried out in Ilala District, stretching from the center of the city to the western outskirts (Figure 1). The district has a population of 1.22 million (2012 census) and is divided into 24 wards. Six wards (Mchikichini, Buguruni, Vingunguti, Ukonga, Majohe and Chanika) were selected for the present study based on their distance to the city centre, population density and environmental characteristics and facilities (Table 1). Mass drug administration with ivermectin and albendazole was implemented in Dar es Salaam by the National LF Elimination Programme in 2006 and 2007. Bed nets for mosquito protection (and insecticides for net impregnation) have been available at subsidized prices to pregnant women and children ≤ 5 years in Dar es Salaam since 2004, whereas permanently impregnated nets were distributed free of charge to every household in 2011 (towards the end of the study period).

**Table 1:** Characteristics of the six wards in Ilala District, Dar es Salaam, included in the study

**Ward**	**Distance from city centre**^**a**^	**Population density**^**b**^	**Brief description**	**Water and sanitary facilities**^**c**^
Mchikichini	3.8 km	21,980	Belongs to old part of city centre. Has of late turned into an area with many high business/residential buildings, but also has many poor concrete houses. Busy business area during daytime.	Some parts with piped water from DAWASCO^d^. Majority rely on bore holes for water. Old underground sewerage system.
Buguruni	6.6 km	21,730	Congested unplanned densely populated residential/commercial area, mainly with poor houses. Some small-scale industries. Established as part of the city in the 1940’s.	No central supply of piped water. Majority rely on privately owned bore holes and shallow wells. DAWASCO also has water kiosks.
Vingunguti	7.1 km	25,570	Congested unplanned densely populated residential/commercial area, mainly with poor houses. Established as industrial and city farming area in 1960’s; later also grew into a residential area.	No central supply of piped water. Private and government owned bore holes, and traditional hand dug shallow wells.
Ukonga	15.2 km	3,490	Mixture of houses, from newly well-built to poor mud houses. Established as part of the city in 1970’s.	Small part with piped water from DAWASCO. Majority rely on private bore holes and shallow wells. Buying of water from vendors common.
Majohe	20.5 km	5,010	Unplanned non-surveyed fast-growing new settlement. Most houses good and well built, though there are few poor ones. Established as part of the city in 1990’s.	No central supply of piped water. Private bore holes and hand dug wells common.
Chanika	30.8 km	470	Peri-urban. With the rapid expansion of city has recently turned into a residential area. Houses ranging from newly built to poor mud houses. Some small-scale farming.	No central supply of piped water. Private and government owned bore holes. Hand dug wells common. Also water from streams.

^a)^ Straight line from Askari Monument to the six study schools.

^b)^ Inhabitants per km^2^, based on 2012 census.

^c)^ Unless otherwise specified, sewerage facilities mainly comprise of open drains, cesspits and septic tanks. Pit latrines of various types are most common sanitary facilities in all six wards.

^d)^ Dar es Salaam Water and Sewerage Company.

### Study design

The study was carried out from March to October 2011 and had a school part and a community part. In the school part, one (or two neighboring, if necessary) public primary schools were selected from each of the six wards, in order to recruit approximately 300 standard one and two pupils from each ward for examination. The included primary schools (PS) were Mivinjeni and Msimbazi Mseto PS in Mchikichini Ward, Buguruni PS in Buguruni Ward, Mtakuja and Kombo PS in Vingunguti Ward, Mzambarauni PS in Ukonga Ward, Majohe PS in Majohe Ward and Chanika and Tungini PS in Chanika Ward. Lists of the pupils were prepared from the school registers, including their name, age, sex and residence. The parents were asked for written consent before further involvement of their children in the study. Examinations started with a short questionnaire administered to each pupil, after which they were requested to provide finger prick blood samples for determination of circulating filarial antigens (CFA) and antibodies to Bm14.

Four of the six wards from the school part were also included in the community part of the study, namely Mchikichini, Vingunguti, Ukonga and Chanika. In each of these, one ten cell unit (smallest administrative unit in Tanzania; comprises of approximately 10–20 neighboring households, each of which may contain many families), from which many of the examined pupils came, was selected. The local leaders were requested for permission to conduct the study, and the study team and leaders agreed on mode of social mobilization and dates for house to house examination of 250–300 community members aged 10 years and above. Due to frequent reluctance from community members to participate in examinations, the survey team in some cases had to modify the sampling method and also include volunteers from 1–2 neighboring ten cell units. At the time of examination, the community members’ name, age, sex and length of stay in Dar es Salaam was recorded, they provided a finger prick blood sample for determination of CFA, they were clinically examined for chronic manifestations related to LF, and a questionnaire was administered to the heads of households. CFA positive individuals were requested to come to a nearby place at night for blood examination for microfilaraemia. The areas around the four communities were inspected for potential vector breeding sites, and safe water, drainage and sewerage facilities were assessed by using a checklist. Vector surveys were carried out in selected households during the peak mosquito season.

### Examination for circulating filarial antigens

Pupils and community members were examined for CFA status by use of Binax NOW Filariasis ICT cards (Inverness Medical Innovations, Maine, USA), according to manufacturers’ instructions and as described [29,30]. Briefly, 100 μl of finger prick blood was drawn into heparinized tubes and transferred to the pad on the ICT card. The card was closed and the result was read after 10 minutes. A few of the tests were invalid, either because the amount of blood obtained was insufficient or because the blood clotted before reaching the reactive area on the test card.

### Examination for antibodies to Bm14

Finger prick blood for examination for antibodies to the recombinant filarial antigen Bm14 was collected from the pupils on TropBio filter paper blood collection discs (TropBio Pty Ltd, Townsville, Australia). Each of the six protrusions on the disc, which have a capacity to absorb 10 μl blood, were saturated with blood from the same pupil. The filter papers were thereafter dried at room temperature for 24 hours, before being placed individually in small zip locked plastic bags and frozen at -20°C. Later, the samples were analyzed in the laboratory for IgG4 antibodies to Bm14 by enzyme linked immunosorbent assay (ELISA) by use of the commercially available CELISA Bm14 kit (Cellabs Pty Ltd, Brokevale, Australia) according to manufacturer’s instructions and as described [31]. Briefly, the samples were thawed and one protrusion from each disc was excised into an Eppendorf tube with 500 μl of sample diluent. The specimens were left in a fridge overnight, and the next morning vortexed to ensure complete extraction of antibodies from the filter paper. From each specimen, 100 μl were transferred to a well in a microtitre plate pre-coated with recombinant Bm14 antigen. Similar volumes of a positive and a negative control from the kit (each in duplicate) and of sample diluent (in six wells; as blanks) were added to wells on the same plate. The plate was incubated for 1 hour at 37°C and thereafter washed four times with PBS/Tween. Enzyme conjugate (100 μl) was added to each well and the plate was incubated at 37°C for 45 minutes. After four washes with PBS/Tween, 100 μl of substrate solution was added to the wells and the plate was incubated in darkness at room temperature for 15 minutes. This was followed by addition of 100 μl of stop solution to each well, after which the optical density (OD) values were read on an ELISA reader (Thermo Scientific Multiskan FC spectrophotometer, Thermo Fischer Scientific Oy, Vantaa, Finland) at dual wavelength of 450/650 nm. OD-values from the positive controls were used to adjust for plate to plate variations. Test specimens with OD-values ≥ 0.4 were considered positive for antibodies to Bm14.

### Examination for microfilariae

Blood from community members was examined for microfilariae by using the counting chamber technique, as previously described [32]. Briefly, 100μl of finger prick blood was drawn into a heparinized capillary tube between 22:00 and 02:00 hours and transferred to labelled microtubes with 1.0 ml of 3% acetic acid. In the laboratory the specimens were transferred to a counting chamber, and the microfilariae were counted under a microscope at 40x magnification. To enhance reliability, samples were counted independently by two different individuals and the mean count was used.

### Clinical examination

During community surveys all study individuals aged 10 years and above were examined for chronic manifestations of LF by a qualified clinician. Manifestations were graded according to size and development stage, but gradings are not used in this presentation.

### Surveys for vector mosquitoes

Adult mosquitoes were collected by battery operated CDC light traps (Model 512; John W. Hock Gainesville, USA) in May-July 2011, during the peak mosquito season. Five houses with poor mosquito proofing measures were selected for this purpose in each of the four study wards. During the first week, mosquitoes were collected from Mchikichini and Chanika wards (i.e. 10 light traps) for 5 nights. The following week the exercise was repeated in Vingunguti and Ukonga wards, and so on, until all wards had been sampled for 3 weeks (i.e. total of 15 sampling nights with 5 traps from each ward). All persons sleeping in the collection room were provided with a 2-mm-mesh polyester un-impregnated bed net and the light trap was placed beside one of the occupied bed nets [14]. Traps were switched on at 18:00 hours and off the next morning at 06:00 hours. Caught mosquitoes were transferred to paper cups covered with netting material. Cotton pads soaked in 10% glucose were placed on top of the cups for feeding the mosquitoes. The cups were transported to the laboratory in a cool box. Live mosquitoes were anaesthetized with diethyl ether, the catch was sorted and the mosquitoes identified on morphological characteristics. Live female mosquitoes were dissected under 20x magnification [14,33]. Wings and legs were removed before head, thorax and abdomen were separated, placed in drops of saline and examined for filarial worms. The mosquitoes were scored as parous or nulliparous after examination of the tracheoles of their ovaries.

### Questionnaire surveys

Two questionnaires were administered during the study: one to the pupils during the school surveys and the other to heads of household during the community surveys. The questionnaires were in Swahili, and primarily focused on socio-economic, behavioral and environmental factors and on knowledge related to LF and its control.

### Ethical considerations

Research and ethical clearance to carry out the study was granted by the Medical Research Coordinating Committee of the National Institute for Medical Research. Permission to conduct the study was moreover requested from all relevant authorities. Request letters were sent to regional and district medical and education officers, as well as to the schools selected for the study. All schools and communities involved in the study were well informed about the study activities and of the importance of them taking part. Written informed consent was requested from all study participants (with parents consenting for individuals below 18 years). Children who did not want to participate, even after consent from their parents, were excluded from the study.

### Data analysis

Data were entered in Excel 2007 and transferred to Stata version 12 for analysis. Geometric mean intensities (GMIs) of Bm14 OD-values were calculated as antilog [(∑logx+1)/*n*]-1, with x being the OD-values and *n* the number of individuals included in the calculation. During the community surveys, when only CFA positive individuals were examined for mf, the community mf prevalence was calculated as: (b/a) × (d/c) × 100, where a = number of individuals in the community examined for CFA, b = number of those examined for CFA being positive, c = number of CFA positives examined for mf, and d = number of those examined for mf being positive [34]. Categorical variables were compared statistically by chi-square (χ^2^) test, whereas continuous variables were compared by t-test or one-way analysis of variance, as appropriate. P-values less than 0.05 were considered statistically significant.

## Results

### School study

A total of 3655 standard 1 and 2 pupils were registered as potential study individuals in the six wards (Table 2). Despite immense efforts to convince parents to enroll their children in the study, many parents did not accept and some children refused after parent’s acceptance. Therefore, only 1697 (46.4%) had a valid test for CFA (range of 265–303 pupils per ward). The mean age of those tested (overall 7.6, range 5–13 years) did not differ significantly between wards (t-test, P > 0.05). The girl to boy ratio (overall 1.0, range 0.8-1.7) was significantly higher in Majohe than in the other wards combined (χ^2^ test, P < 0.001).

**Table 2:** Circulating filarial antigens (CFA) and antibodies to Bm14 as seen in the school study

**Ward**	**No. children registered**	**CFA**				**Bm14**			
		**No. tested**^**a **^**(% of registered)**	**Mean age in years (range)**	**Girl:boy ratio**	**No. tests positive (%)**	**No. tested (% of registered)**	**No. tests positive (%)**	**GMI**^**b **^**for all examined**	**GMI**^**b **^**for positives only**
Mchikichini	347	276 (79.5)	7.5 (5–12)	0.9	7 (2.5)	271 (78.1)	20 (7.0)	0.1474	0.7478
Buguruni	582	286 (49.1)	7.6 (5–11)	1.0	7 (2.4)	270 (46.4)	9 (3.3)	0.1249	1.5498
Vingunguti	782	303 (38.7)	7.5 (5–12)	1.1	16 (5.3)	298 (38.1)	17 (5.7)	0.1445	0.9454
Ukonga	692	299 (43.2)	7.4 (5–11)	0.8	3 (1.0)	286 (41.3)	7 (2.5)	0.0847	1.1687
Majohe	700	268 (38.3)	7.7 (6–12)	1.7	12 (4.5)	242 (34.6)	18 (7.4)	0.1368	1.1817
Chanika	552	265 (48.0)	7.8 (5–13)	1.0	5 (1.9)	255 (46.2)	10 (3.9)	0.0962	1.0394
Total	3655	1697 (46.4)	7.6 (5–13)	1.0	50 (3.0)	1622 (44.4)	81 (5.0)	0.1223	1.0338

The pupils were from primary schools located in six wards in Ilala District, Dar es Salaam. All pupils tested for antibodies to Bm14 were also tested for CFA.

^a)^ Excluding 29 children with invalid tests (9, 4, 5, 2, 2, 7 from Mchikichini to Chanika, respectively).

^b)^ Geometric mean intensity in OD-values.

**Figure 2:**
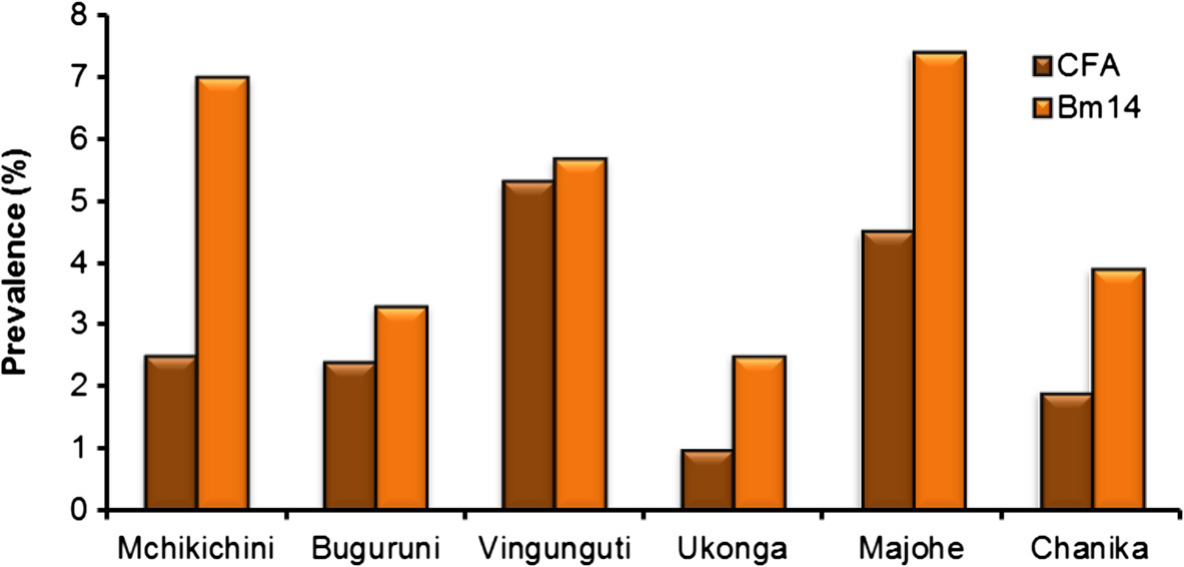
**Prevalence of circulating filarial antigens (CFA) and antibodies to Bm14 as seen in the school study.** The pupils were from primary schools located in six different wards in Ilala District, Dar es Salaam.

**Figure 3:**
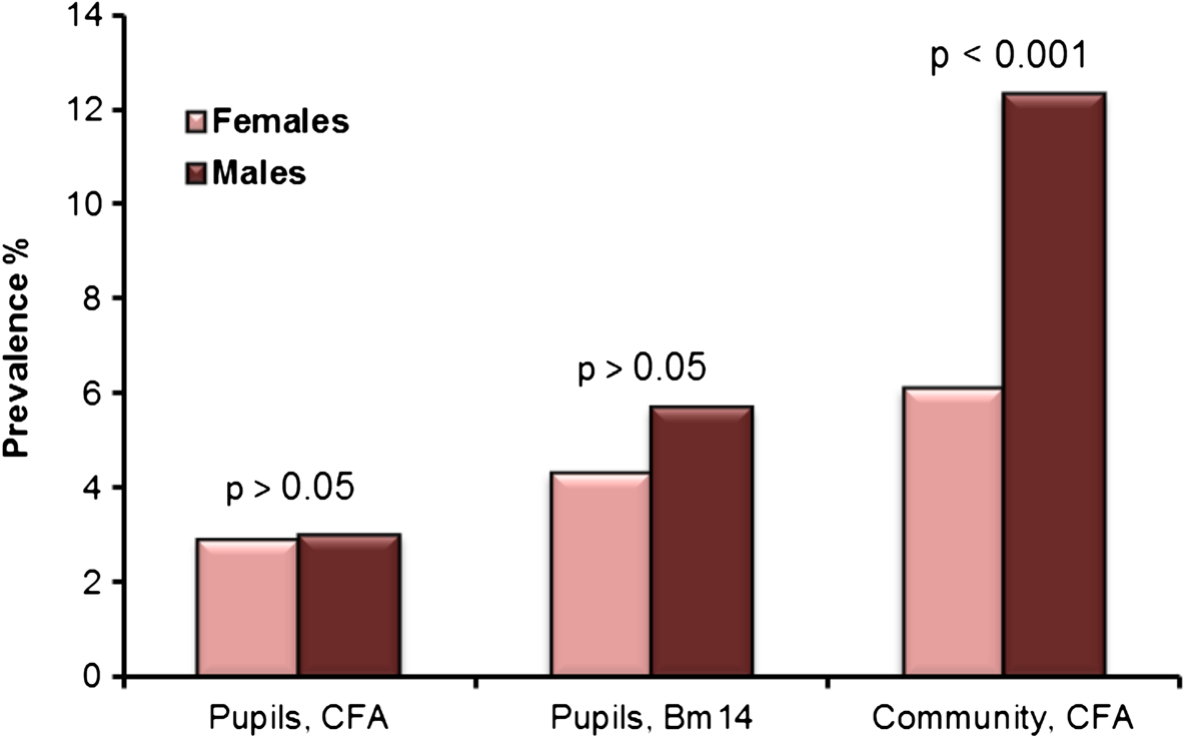
**Gender specific prevalence of circulating filarial antigens (CFA) and antibodies to Bm14.** Shown for all examined pupils combined (school study) and all examined community members combined (community study). Differences were tested by χ^2^ test.

Fifty pupils were positive for CFA, giving an overall prevalence of 3.0% (ward range 1.0-5.3%; Table 2, Figure 2). Prevalences were significantly higher in Vingunguti (5.3%) and Majohe (4.5%) than in the other four wards combined (2.0%; χ^2^ test, P = 0.001 and 0.016, respectively). There was no significant difference in prevalence of CFA between girls and boys, neither overall (Figure 3) nor in the individual wards (χ^2^ test, P > 0.05 for all tests).

Most pupils examined for CFA (95.5%), were also examined for antibodies to Bm14. Among them, 81 (5.0%) tested positive (ward range 2.5-7.4%; Table 2, Figure 2). Prevalences were significantly higher in Majohe (7.4%), Mchikichini (7.0%) and Vingunguti (5.7%) than in the other three wards combined (3.2%; χ^2^ test, P = 0.004, 0.003 and 0.056, respectively). There was no significant differences in prevalence of antibodies to Bm14 between girls and boys, neither overall (Figure 3) nor in the individual wards (χ^2^ test, P > 0.05 for all tests). The same pattern was seen for OD-value GMIs calculated on the basis of all examined, with significantly higher mean levels for Majohe, Mchikichini and Vingunguti than for the other three wards combined (t-test, P = 0.02, 0.001 and < 0.001, respectively) and no significant difference between boys and girls (t-test, P > 0.05 for all tests). In all wards, the prevalence of antibodies to Bm14 was higher than the prevalence of CFA.

Among pupils with a valid CFA test, 1496 (88.2%) replied to the questionnaire (Table 3). 99.2% indicated they had been born in the city. Possession of a TV or a fridge by their family was used as an indicator of economic wealth. Significantly more pupils from Buguruni, Mchikichini and Ukonga combined than from Vingunguti and Majohe combined came from families that had a TV (69.5% vs. 49.3%; χ^2^ test, P < 0.001) or a fridge (48.4% vs. 24.0%; χ^2^ test, P < 0.001). Interestingly, the CFA prevalence was significantly higher in the latter than the former group of wards (5.0% vs. 2.1%; χ^2^ test, P = 0.005) thus suggesting an inverse relationship between ward wealth and CFA prevalence. Chanika ward did not fit this pattern, perhaps because of its more rural environment. When the relationship between wealth and CFA was analyzed on an individual level, the prevalence of CFA did not differ significantly between pupils from families with or without the two wealth indicators, neither for the five most urban wards combined (3.2% vs. 3.1% for TV; 2.2% vs. 3.8% for fridge; χ^2^ test, P > 0.05) nor for Vingunguti and Majohe wards combined (6.3% vs. 3.7% for TV; 5.2% vs. 4.9% for fridge; χ^2^ test, P > 0.05).

**Table 3:** Questionnaire-based survey in the school study

**Question**	**Response**	**Ward**^**a**^						
		**Total**	**Mchikichini**	**Buguruni**	**Vingunguti**	**Ukonga**	**Majohe**	**Chanika**
		**(n=1496)**	**(n=267)**	**(n=257)**	**(n=288)**	**(n=247)**	**(n=195)**	**(n=242)**
Were you born in Dar es Salaam?	Yes	1484 (99.2)	261 (97.8)	257 (100.0)	287 (99.7)	246 (99.6)	194 (99.4)	239 (98.8)
Does your family own a TV set?	Yes	857 (57.3)	182 (68.2)	184 (71.6)	160 (55.6)	170 (68.8)	78 (40.0)	83 (34.3)
Does your family own a fridge?	Yes	528 (35.3)	132 (49.3)	128 (49.8)	66 (22.9)	113 (45.7)	50 (25.6)	39 (16.1)
Which of these infections is transmitted by mosquitoes (select only one)?	None	160 (10.7)	47 (17.6)	27 (10.5)	25 (8.7)	18 (7.3)	13 (6.7)	30 (12.4)
	Cholera	296 (19.8)	71 (26.6)	33 (12.8)	64 (22.2)	54 (21.9)	31 (15.9)	43 (17.8)
	Worms^b^	288 (19.3)	37 (13.9)	50 (19.5)	53 (18.4)	55 (22.3)	39 (20.0)	54 (22.3)
	Schistosomiasis	257 (17.2)	34 (12.7)	28 (10.9)	50 (17.4)	38 (15.4)	31 (15.9)	76 (31.4)
	LF	254 (17.0)	52 (19.5)	63 (24.5)	24 (8.3)	46 (18.6)	54 (27.7)	15 (6.2)
	HIV/AIDS	241 (16.1)	26 (9.7)	56 (21.8)	72 (25.0)	36 (14.6)	27 (13.8)	24 (9.9)
Do you sleep under a bed net?	No	108 (7.2)	19 (7.1)	30 (11.7)	19 (6.6)	16 (6.5)	6 (3.1)	18 (7.4)
	Yes, occasionally	230 (15.4)	52 (19.5)	60 (23.3)	28 (9.7)	29 (11.7)	31 (15.9)	30 (12.4)
	Yes, always	1158 (77.4)	196 (73.4)	167 (65.0)	241 (83.7)	202 (81.8)	158 (81.0)	194 (80.2)

The pupils were from primary schools located in six wards in Ilala District, Dar es Salaam. Table shows the number (%) of pupils who gave the indicated response to the indicated question.

^a)^ Female to male ratio was 0.9, 1.1, 1.1, 0.9, 1.9 and 1.0, and mean age (range) in years was 7.5 (5–12), 7.7 (5–11), 7.5 (5–12), 7.4 (5–11), 7.7 (6–11) and 7.8 (5–13), respectively, among those interviewed in Mchikichini, Buguruni, Vingunguti, Ukonga, Majohe and Chanika.

^b)^ Intestinal worms.

The knowledge about transmission of the infectious diseases listed in the questionnaire was low (Table 3). Only a few pupils knew that LF was transmitted by mosquitoes (overall 17.0%; ward range 6.2-27.7%), and no clear relationship appeared between ward level of this knowledge and CFA prevalence. The majority of pupils indicated that they slept under a bed net (77.4% always, 15.4% occasionally), and commonly stated that this was mainly due to the mosquito nuisance in their homes. No clear relationship was seen between bed net use and CFA or Bm14 prevalence, neither overall or in individual wards, probably because of the high level of bed net coverage.

### Community study

A total of 1212 community members aged 10 years and above were tested for CFA in the four wards (Table 4). The mean age of those tested was 33.3 years, and the female to male ratio was 0.8. Both of these indices differed significantly between wards (one-way, P < 0.001; χ^2^ test, P < 0.001). 84.7% indicated they had stayed more than 5 years in the city.

**Table 4:** Circulating filarial antigens (CFA) and microfilaraemia as seen in the community study

**Ward**	**CFA**					**Microfilaraemia**		
	**No. tested**^**a**^	**Mean age in years (range)**	**Female:male ratio**	**No. staying ≥ 5 years in the city (%)**	**No. tests positive (%)**	**No. tested**	**No. positive (%)**	**Community mf prevalence**^**b **^**in %**
Mchikichini	287	29.7 (10–81)	0.6	252 (87.8)	20 (7.0)	15	2 (13.3)	0.9
Vingunguti	348	36.2 (10–92)	0.6	326 (93.7)	47 (13.5)	39	3 (7.7)	1.0
Ukonga	293	32.6 (10–88)	1.1	243 (82.9)	23 (7.8)	21	2 (9.5)	0.7
Chanika	284	34.2 (10–80)	1.4	205 (72.2)	25 (8.8)	24	1 (4.2)	0.4
Total	1212	33.3 (10–92)	0.8	1026 (84.7)	115 (9.5)	99	8 (8.1)	0.8

Community members (≥ 10 years) were from four wards in Ilala District, Dar es Salaam. Only CFA positive individuals were tested for microfilaraemia.

^a)^ Excluding seven individuals with invalid tests (all from Ukonga).

^b)^ See Methods for calculation.

**Figure 4:**
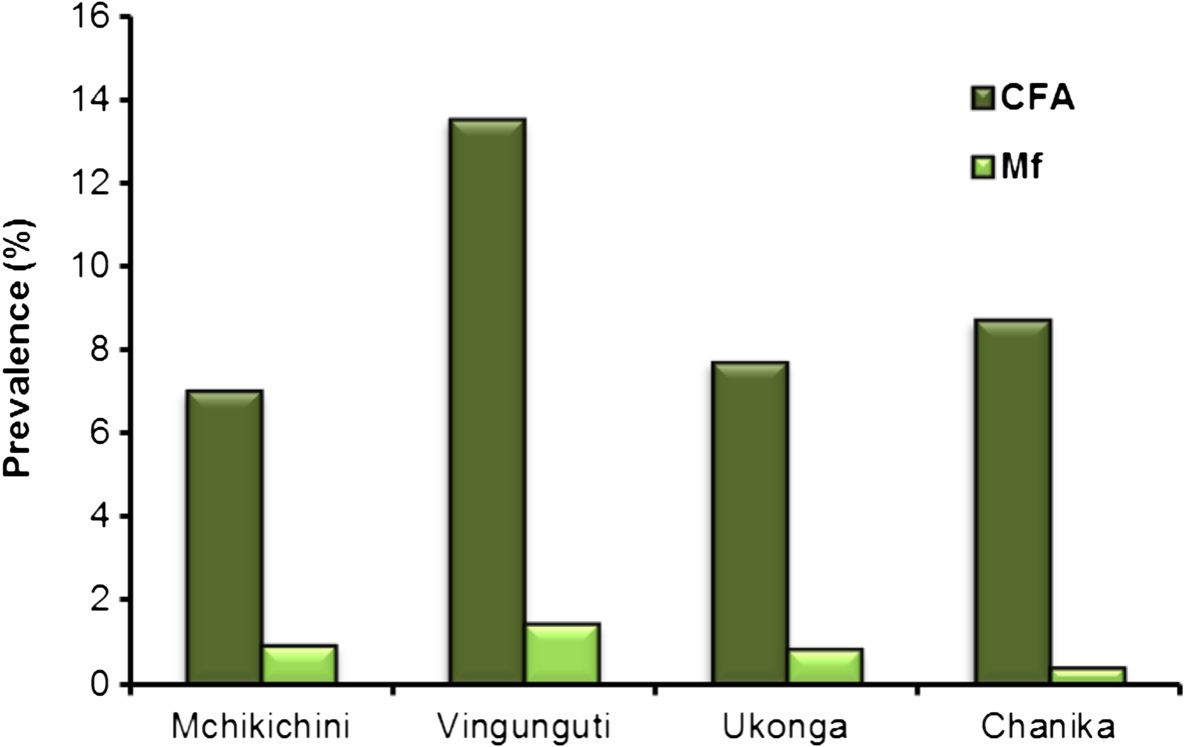
**Prevalence of circulating filarial antigens (CFA) and microfilaraemia as seen in the community study.** Community members were from four different wards in Ilala District, Dar es Salaam.

One hundred and fifteen individuals were CFA positive, giving an overall prevalence of 9.5% (ward range 7.0-13.5%; Table 4, Figure 4). The prevalence was significantly higher in Vingunguti (13.5%) than in the other three wards combined (7.9%; χ^2^ test; P < 0.002). Overall, the CFA prevalence was significantly higher in males than in females (12.3% vs. 6.1%; χ^2^ test, P < 0.001; Figure 3), and it was significantly higher in individuals aged 25 years and above than in younger individuals (10.9% vs. 6.6%; χ^2^ test, P = 0.017).

Of the 115 individuals found positive for CFA, 99 were also tested for mf (Table 4). Eight of these were mf positive (8.1%; ward range 4.2-13.3%; numbers too low for statistical testing of ward differences). Six of the positives were males and 2 were females (overall mean age 39.3 years, range 13–73 years). As only CFA positives were targeted for mf testing, and as not all of these turned up for night blood sampling, an equation was used to calculate the approximate mf prevalence for the community members on the basis of available data (see Methods). This gave an overall community mf prevalence of 0.8% (ward range 0.4-1.0%; highest mf prevalence in Vingunguti; Figure 4).

The 1212 community members examined for CFA were also examined for chronic manifestations of LF (Table 5). Twenty six (2.1%) had elephantiasis (15 females, 11 males), with no significant overall difference in prevalence between females and males (2.7% vs. 1.7%; χ^2^ test, P > 0.05). Among the 659 males, 118 (17.9%) had hydrocele.

**Table 5:** Chronic clinical manifestations of lymphatic filariasis as seen in the community study

**Ward**	**Females**		**Males**		**No. males with hydrocoele (%)**	**No. females and males with elephantiasis**^**a **^**(%)**
	**No. examined**	**Mean age in years (range)**	**No. examined**	**Mean age in years (range)**		
Mchikichini	109	30.9 (11–81)	178	29.0 (10–78)	10 (5.6)	2 (0.7)
Vingunguti	129	34.9 (10–85)	219	37.0 (10–92)	36 (16.4)	9 (2.6)
Ukonga	151	29.4 (10–75)	142	36.0 (10–88)	39 (27.5)	5 (1.7)
Chanika	164	33.4 (10–80)	120	35.3 (10–79)	33 (27.5)	10 (3.5)
Total	553	32.1 (10–85)	659	34.3 (10–92)	118 (17.9)	26 (2.1)

Community members (≥ 10 years) were from four wards in Ilala District, Dar es Salaam.

^a)^ 0, 5, 3 and 7 females and 2, 4, 2 and 3 males from Mchikichini, Vingunguti, Ukonga and Chanika, respectively, had elephantiasis.

One hundred and seventy two of the examined community members were heads of households and responded to the questionnaire (ward range 38–49; Table 6). The answers revealed differences in main occupation, household income and educational level between the wards. Thus, in Vingunguti, which had the highest CFA prevalence, the population was mainly small-scale traders and self-employed (94.7%), with low income (81.6%) and only few had completed secondary school (10.5%). Mchikichini and Ukonga had more employees (28.6-30.8%), the proportion with high income was higher (53.1-59.0%) and more had completed secondary school (32.7-33.3%). The most peripheral ward of Chanika had many peasants (47.8%), many with low income (73.9%) and few had completed secondary school (8.7%). Overall, less than half of the respondents related elephantiasis and hydrocele to mosquito bites (40.1% and 29.1%, respectively), the majority indicated they slept under bed nets (93.0%), and very few indicated they had taken drugs for LF during at least one of the two MDA campaigns in 2006 and 2007 (18.6%).

**Table 6:** Q**uestionnaire-based survey among heads of households in the community study**

**Question**	**Response**	**Ward**^**a**^				
		**Total (n=172)**	**Mchikichini (n=49)**	**Vingunguti (n=38)**	**Ukonga (n=39)**	**Chanika (n=46)**
Your main occupation?	Employee	33 (19.2)	14 (28.6)	2 (5.3)	12 (30.8)	5 (10.9)
	Business/self employed	105 (61.0)	32 (65.3)	36 (94.7)	20 (51.3)	17 (37.0)
	Peasant	24 (14.0)	0 (0.0)	0 (0.0)	2 (5.1)	22 (47.8)
	Other	10 (5.8)	3 (6.1)	0 (0.0)	5 (12.8)	2 (4.3)
Your educational level?	Not completed primary	31 (18.0)	4 (8.2)	3 (7.9)	7 (17.9)	17 (37.0)
	Completed primary, but not secondary	104 (60.5)	29 (59.2)	31 (81.6)	19 (48.7)	25 (54.3)
	Completed secondary	37 (21.5)	16 (32.7)	4 (10.5)	13 (33.3)	4 (8.7)
Average monthly income of your household?	< 80,000 TSh	104 (60.5)	23 (46.9)	31 (81.6)	16 (41.0)	34 (73.9)
	≥ 80,000 TSh	68 (39.5)	26 (53.1)	7 (18.4)	23 (59.0)	12 (26.1)
What causes elephantiasis?	Mosquito bite	69 (40.1)	24 (49.0)	17 (44.7)	10 (25.6)	18 (39.1)
	Other^b^	11 (6.4)	1 (2.0)	3 (7.9)	2 (5.1)	5 (10.9)
	Do not know	92 (53.5)	24 (49.0)	18 (47.4)	27 (69.2)	23 (50.0)
What causes hydrocele?	Mosquito bite	50 (29.1)	15 (30.6)	19 (50.0)	6 (15.4)	10 (21.7)
	Other^b^	8 (4.7)	1 (2.0)	3 (7.9)	1 (2.6)	3 (6.5)
	Do not know	114 (66.3)	33 (67.3)	16 (42.1)	32 (82.1)	33 (71.7)
Do you sleep under bed net?	Yes	160 (93.0)	47 (95.9)	35 (92.1)	37 (94.9)	41 (89.1)
Have you ever taken drugs for LF?	Yes	32 (18.6)	5 (10.2)	7 (18.4)	10 (25.6)	10 (21.7)

Participants were from four wards in Ilala District, Dar es Salaam. Table shows the number (%) of individuals who gave the indicated response to the indicated question.

^a)^ Female to male ratio was 0.6, 0.3, 1.3 and 0.4, and mean age (range) in years was 42.9 (21–75), 36.7 (18–73), 42.9 (18–80) and 46.5 (26–79), respectively, among those interviewed in Mchikichini, Vingunguti, Ukonga and Chanika wards.

^b)^ Other choices were: witchcraft, eating bad food, touching or sleeping with person with the mentioned sign, Gods decision, injury, and stepping barefooted on dirty matter.

A total of 12096 vector mosquitoes were caught in the light traps (Table 7). The great majority were *Cx. quinquefasciatus* (99.0%), followed by a few *An. gambiae* (0.9%) and *An. funestus* (0.1%). The two anophelines were almost exclusively found in the most peripheral ward of Chanika. The mosquito density, measured as numbers caught per trap night, was highest in the most central ward of Mchikichini, and lowest in Chanika. Of the 4522 dissected mosquitoes, none had infective larvae and only one *Cx. quinquefasciatus* had immature filarial larval stages (one L1 larva and one L2 larva). The majority of dissected *Cx. quinquefasciatus* in all wards were nulliparous (overall 61%).

**Table 7:** Light trap collection of lymphatic filariasis vectors in the community study

**Ward**	**No. trap nights**^**a**^	**Species**	**No. caught**			**No. caught/trap night**	**No. dissected**	**No. infected**
			**Total**	**Dead**	**Alive**			
Mchikichini	71	*An. gambiae*	1	1	0	-	0	0
		*An. funestus*	0	0	0	-	0	0
		*Cx. quinq.*	4420	2561	1859	-	1631^b^	1^c^
		All	4421	2562	1859	62.3	1631	1
Vingunguti	69	*An. gambiae*	0	0	0	-	0	0
		*An. funestus*	0	0	0	-	0	0
		*Cx. quinq.*	2939	1827	1112	-	1054^b^	0
		All	2939	1827	1112	42.6	1054	0
Ukonga	70	*An. gambiae*	2	2	0	-	0	0
		*An. funestus*	0	0	0	-	0	0
		*Cx. quinq.*	3733	2274	1459	-	1456^b^	0
		All	3735	2276	1459	53.4	1456	0
Chanika	68	*An. gambiae*	102	62	40	-	40	0
		*An. funestus*	10	7	3	-	3	0
		*Cx. quinq.*	889	548	341	-	336^b^	0
		All	1001	617	384	14.7	379	0
Total	-	*An. gambiae*	105	65	40	-	40	0
		*An. funestus*	10	7	3	-	5	0
		*Cx. quinq.*	11981	7210	4771	-	4477^b^	1
		All	12096	7282	4814	43.5	4522	1

Mosquitoes were trapped in houses in the four wards in Ilala District, Dar es Salaam.

^a)^ Few times inhabitants were not at home on trapping nights.

^b)^ 63.8%, 60.3%, 57.3% and 62.2% were nulliparous (had not yet taken a blood meal) in Mchikichini, Vingunguti, Ukonga and Chanika, respectively (overall 60.8%).

^c)^ One mosquito had one L1 and one L2 larvae.

## Discussion

The present study examined for LF in a transect of Dar es Salaam, ranging from sites near the center to the semi-rural periphery about 30 km away. It covered localities with a range of different population densities, socioeconomic and environmental characteristics, and water, sewerage and sanitary facilities, being representative for the majority of inhabitants of Dar es Salaam. The more affluent areas (mainly in the center and in the northern part of the city) were not covered, as the likelihood of finding LF infection and disease in these was considered small. The great majority of study individuals were permanent residents in the city, with 99% of pupils indicating they had been born there and 85% of community members indicating they had stayed there for more than 5 years.

As opposed to experience from previous studies carried out by the survey team in rural areas, LF research in the urban settings provided numerous challenges, as also reported by others [21,35]. Despite great efforts to engage and motivate individuals to take part in the study, the team was frequently met by reluctance and mistrust. In the primary schools, only half of the registered children took part in the study, a major reason being that the parents refused to sign the consent forms. Many parents were not even prepared to discuss advantages and disadvantages of their children’s involvement with the study team. Some parents suspected that the children would be tested for HIV, despite assurance that this would not happen. Some children refused examination despite their parents’ consent, and in Majohe many of the boys ran away each time the survey team came to carry out examinations. These refusals are not likely to have significantly affected the overall prevalence of the measured markers in the children. During the house to house community surveys many individuals also refused to take part, despite involvement of local leaders (including religious) in recruitment. Common excuses were their busy schedule, preconceived ideas relating blood sampling to HIV testing, and requests for payment. During the community surveys in Mchikichini and Vingunguti it was necessary, as a last resort, to change methodology and include volunteers from nearby ten cell units in the survey. This may have attracted a higher than average proportion of individuals with clinical manifestations, in the expectation that they would get some relief from the examinations.

At some of the sites, these events affected the gender balance, with significantly more girls than boys being examined in school surveys in Majohe, and significantly more males than females examined in community surveys in Mchikichini and Vingunguti. In semi-rural Chanika more females than males were included because many males were working more central in the city. As it is well known from most endemic areas that the LF prevalence is higher in males than females [20,36,37], especially among adults, the gender balance should be kept in mind when interpreting the findings.

In the school surveys, prevalences of CFA (a marker of adult *W. bancrofti* infection [30]) and antibodies to Bm14 (a marker of exposure to *W. bancrofti*[38]) were generally low, indicating that the level of recent and ongoing LF transmission in the study areas was low. Much higher prevalences of CFA have been reported from Standard 1 pupils in rural endemic areas of coastal Tanzania [39]. The levels of both markers were higher in children from Vingunguti and Majohe than in children from the other examined wards, probably as result of an abundance of waste water ponds, blocked ditches and drains and poor quality pit latrines, favoring breeding of Culicine vectors, in these areas. When using possession of a TV or fridge as indicators of family wealth, pupils from Vingunguti and Majohe moreover came from relatively less wealthy families. Thus, as reported from other urban studies [35,40-42], there appeared to be an overall trend of higher LF prevalence in urban areas with lower socioeconomic status. A relatively high prevalence of antibodies to Bm14 despite low CFA prevalence in Mchikichini suggests a recent increase in transmission in this ward. It is likely that a major boom in density of *Cx. quinquefasciatus* during the time of the study [43], originating from the nearby swampy Msimbazi valley and receiving much public and press attention, had boosted the LF transmission.

Higher infection prevalences were, as expected, seen in the community surveys, but prevalences were still low when compared to the levels commonly seen in endemic rural areas of coastal Tanzania [13,18-20], and to those reported from Dar es Salaam in the past [28]. Among the four examined wards, the CFA prevalence was again highest in Vingunguti (populated mainly by petty traders with low educational level and income). Very few community individuals had mf in the blood (0.8% overall community prevalence), meaning a limited source of infection for the vectors, and thereby limited transmission. Although LF infection prevalences were relatively low, chronic disease manifestations were common. There was a tendency for chronic disease to be more common in the peripheral wards (Ukonga, Chanika), especially for hydrocele, in particular when considering that in the two more central wards individuals with manifestations may have been attracted to volunteer for examination. The high prevalence of chronic manifestations suggests that infections were previously much more common, and that much of what was seen during the survey was irreversible chronic disease induced in the past. Thus, despite the low level of infection seen, there is still a great need for chronic disease management, especially in the elder part of the community population.

The school study showed no significant difference in CFA prevalence between girls and boys, which is in agreement with previous observations on schoolchildren of same age [39]. Similarly, and interestingly, gender differences in prevalence of antibodies to Bm14 were not seen in these children, suggesting a similar level of exposure among girls and boys in this age group. In the adults, on the other hand, the prevalence of CFA was significantly higher in males than in females, and 6 of the 8 identified mf positive individuals were males. This commonly observed higher LF infection prevalence among adult males has been suggested to stem from behavioral and/or physiological differences between the genders [20,36,37]. The present study did not point to gender differences in exposure during childhood as a reason for gender differences in LF infection prevalence in adults.

In addition to suggest an association between LF and low socioeconomic and educational status, the questionnaires indicated that knowledge about LF was generally low at all study sites and among both children and adults. Thus, only 17% of pupils pointed to LF as a mosquito transmitted infection, and only 40% and 29% of adults knew that manifestations of hydrocele and elephantiasis, respectively, were related to mosquito bites. The use of bed nets, on the other hand, was common at all sites among both children and adults, mainly to protect against mosquito nuisance and malaria. On the question of taking drugs for LF (during one or both of the two previous MDAs), the answers among the community members were in sharp contrast to the treatment coverage figures reported for Ilala District by the National LF Elimination Programme (87% of total population in 2006, 74% in 2007), with only 18.6% of them indicating they had taken the drugs. Only 302 (17.8%) and 109 (6.4%) of the 1697 pupils included in the study were 5 years and above during the MDAs in 2007 and 2006, respectively, and could potentially have been treated.

During the vector surveys, large numbers of *Cx. quinquefasciatus* were caught at all sites, a finding resembling those from other recent surveys in the city [10,44]. Dar es Salaam has had intensive malaria vector control for many years and very few *Anopheles* mosquitoes were caught mainly in the periphery of the city. The survey team observed plenty of breeding sites for *Cx. quinquefasciatus* in the study wards, ranging from waste water pools and blocked open drains to poor quality pit latrines, cesspits and septic tanks, as previously reported [10]. During the rainy season, contaminated spill water was seen in most of the study areas, but particularly in Vingunguti and Mchikichini. The mentioned boom in *Cx. quinquefasciatus* breeding in the Msimbazi valley during the time of the study resulted in the largest density of these vectors caught in Mchikichini. None of the dissected mosquitoes had infective larvae and only one mosquito was infected with immature larval stages. This may be partly a reflection of the low microfilariae prevalence in the human population, but also of the low age of the vector population. A high proportion of the dissected *Cx. quinquefasciatus* were nulliparous, meaning they had not yet taken a blood meal. The apparent general short lifespan of these vectors in the urban environment probably makes them rather inefficient in transmitting LF unless they are present in extremely high numbers [41,45,46].

LF is well known to occur with high prevalence in rural coastal Tanzania (e.g. [13,18-20]). Past surveys in Dar es Salaam also indicated high prevalences of LF. Night blood surveys among males in Dar es Salaam showed mf prevalences of 29% [25], 37% [26] and 16% [27], and Minjas and Kihamia [28] reported mf prevalences in Dar es Salaam to be 15-25% in adults above 30 years. This is in strong contrast to the present study, during which an overall mf prevalence of less than 1% was observed in the adult population. CFA prevalences are generally considerably higher than mf prevalences [20], but also the CFA prevalences in the present study were low (overall 9.5% for adults and 3.0% for schoolchildren). A number of factors may be responsible for this remarkable decrease: 1. The ongoing Urban Malaria Control Programme has considerably reduced the number of *Anopheles* mosquitoes, which in addition to being vectors of malaria are also very efficient vectors of LF in Sub-Saharan Africa; 2. Many areas previously covered with surface water, swamps and vegetation, providing habitats for *Anopheles sp.* breeding, have dried up due to building activities. 3. *Cx. quinquefasciatus* mosquitoes are breeding profoundly in most areas of Dar es Salaam and are currently the most important vectors of LF in the city. However, these appear to be rather short-lived in the urban environment, and only where they occur in enormous numbers will enough survive long enough to be able to maintain intense and effective transmission. 4. Bed nets are widely used, and together with mosquito screening of houses and generally improved house constructions, have reduced exposure to mosquitoes; 5. MDA with a combination of ivermectin and albendazole was implemented twice (2006 and 2007) in Dar es Salaam by the National LF Elimination Programme, although with many logistic challenges. Thus, despite the fact that rapid urbanization in areas with hot and humid climates has often been seen to sustain or even increase transmission of LF [22], the overall situation appears to be different in Dar es Salaam, probably due to a favorable combination of the above mentioned factors.

## Conclusion

The study indicated that a marked decrease in the burden of LF infection had occurred in the metropolis of Dar es Salaam during recent years, simultaneously with a rapid growth of the population and expansion of areas with poor water, drainage and sanitation facilities. The observed findings are encouraging and give hope that integration of different methods and efforts in fighting LF in urban environments can be successful. However, it was clear from the study that although the burden of infection had decreased, there are still infected individuals and ongoing transmission, in particular in areas where less privileged populations are residing. Improving the awareness among people about the role of mosquitoes in the transmission of LF, more thorough implementation of environmental sanitation to reduce *Cx. quinquefasciatus* breeding, and continued MDA to high-risk areas, will be important measures to further reduce and perhaps eliminate the transmission of LF. It was also clear from the study that the burden of chronic disease is still considerable, especially in the elderly part of the population, and that facilities and resources for disease management should be made available to those affected. The development has so far been promising, but continuous efforts are necessary to ensure elimination of LF as a public health problem.

## Competing interests

The authors declare that they have no competing interests.

## Authors’ contributions

MEM, MNM, EMP, FWM and PES conceived and designed the study. MEM collected the field data under supervision of EMP, FWM and PES. MEM performed the laboratory tests, analyzed the data and drafted the manuscript under the supervision of PES. All authors read and approved the final manuscript.
